# Deformation Modes and Anisotropy of Anti-Perovskite Ti_3_AN (A = Al, In and Tl) from First-Principle Calculations

**DOI:** 10.3390/ma10040362

**Published:** 2017-03-29

**Authors:** Kuankuan Chen, Cong Li, Meng Hu, Xun Hou, Chunmei Li, Zhiqian Chen

**Affiliations:** Faculty of Materials and Energy, Southwest University, Chongqing 400715, China; chenkuan2561@email.swu.edu.cn (K.C.); licongchn@163.com (C.L.); humeng@email.swu.edu.cn (M.H.); hou_xunyx@163.com (X.H.); lcm1998@swu.edu.cn (C.L.)

**Keywords:** deformation mode, anisotropy, thermal conductivity, first-principle calculations

## Abstract

Deformation modes were studied for Ti_3_AN (A = Al, In and Tl) by applying strain to the materials using first-principle calculations. The states of the bonds changed during the deformation process, and the Ti-N bonds remained structurally stable under deformation. The elastic anisotropy, electronic structures, hardness, and minimum thermal conductivity of anti-perovskite Ti_3_AN were investigated using the pseudo potential plane-wave method based on density functional theory. We found that the anisotropy of Ti_3_InN was significantly larger than that of Ti_3_AlN and Ti_3_TlN. All three compounds were mechanically stable. The band structures of the three compounds revealed that they were conductors. The minimum thermal conductivities at high temperature in the propagation directions of [100], [110], and [111] were calculated by the acoustic wave velocity, which indicated that the thermal conductivity was also anisotropic. It is indicated that Ti_3_InN is a good thermal barrier material.

## 1. Introduction

Ceramic materials have excellent properties, such as high hardness, good high-temperature performance, corrosion resistance, and oxidation resistance [1]. Meanwhile, metal materials also have outstanding properties, including good electrical and thermal conductivity [2]. However, certain limitations in applications exist for both types of materials. A material called “cermet” has been developed to overcome these limitations. Comprehensive studies on cermet have provided a scientific and technological basis for their application [3]. Cermet has the form M*_n_*_+1_AX*_n_*, where M represents early transition metals, A represents A-group elements, and X is either C or N. Nowotny et al. [4] reported around one hundred mono-phase ternary nitrides and carbides, such as M_2_AX, M_3_AX_2_, and M_4_AX_3_. In addition, advanced M*_n_*_+1_AX*_n_* materials, such as M_5_AX_4_, M_6_AX_5_, and M_7_AX_5_, have been proven to exist in subsequent studies [5,6,7].

Recently, a crystal structure was characterized for the M_3_AlX phase (M = Ti, Zr or Hf and X = C or N), which is a cubic structure with space group $\text{Pm}\bar{3}\text{m}$, such as cubic Ti_3_AlN and cubic Ti_3_AlC [8,9,10]. The structure is known as an anti-perovskite [11], which is a special type of perovskite having a metallic face-centered cubic structure with C or N atoms occupying the body-centered positions. Ternary carbides or nitrides with an anti-perovskite structure possess a wide range of interesting properties due to their chemical composition [12,13,14]. Anti-perovskite nitrides have captivating electronic properties and can be tuned to be conductors, insulators, or semiconductors. In addition, this is a relatively unexplored branch of the perovskite family [15,16]. Sr_3_AsN, Sr_2_SbN, and Sr_3_BiN are semiconductors with band gaps of 0.49, 0.31, and 0.26 eV, respectively [17], and Ca_3_GeN, Ca_3_SnN, and Ca_3_PbN are conductors [18]. The superconductivity of anti-perovskite structural compounds has been studied previously, and superconductivity has been observed for Ni_3_MgC and Ni_3_CdC [19,20]. In addition, some anti-perovskite structural compounds possess good mechanical properties, such as Sc_3_AlC, Sc_3_AlN, and Sc_3_InN [4,21,22]. Researchers have shown interest in the multifarious and special properties of anti-perovskites, and the available reports clearly emphasize the need to explore such compounds.

It is useful to explore Ti-based anti-perovskite carbides and nitrides from different perspectives. Ti_3_AlN was reported to have excellent properties and was predicted to be a novel damage-tolerant nitride [3]. The elements In and Tl are in the same A group as Al; thus, it is easy to assume that Ti_3_InN and Ti_3_TlN may manifest similar properties. Djellal Cherrad et al. [23] investigated the electronic structure and bonding properties of anti-perovskite Ti_3_AN (A = Al, In, and Tl). Unfortunately, the mechanical deformation modes, hardness, and minimum thermal conductivity at high temperature are not completely clear, and this deficiency impedes further study of Ti_3_AN. Hence, it is necessary to conduct further study on Ti_3_AN to predict its important physical properties and to study the relationships between the various properties. This is advantageous for the use of these compounds in practical applications.

## 2. Calculations

### 2.1. Calculation Parameters

First-principle methods have been widely used to investigate material properties. In addition, these methods have shown excellent accuracy in the study of many properties for numerous materials [24]. First-principle calculations were performed with the CASTEP program based on density functional theory (DFT) [25,26]. We used the Ceperley-Alder-Perdew-Zunger (CA-PZ) method under the local density approximation (LDA) to describe the electronic exchange-correlation terms [27,28]. Interactions of electrons with the ion cores were represented by the Vanderbilt-type ultra-soft pseudopotential. The wave-function was expanded by the plane-wave basis set under periodic boundary conditions. The outer electronic configuration considered in the calculation of the atomic pseudopotentials was as follows: Ti: 3s^2^3p^6^3d^2^4s^2^, Al: 3s^2^3p^1^, In: 4d^10^5s^2^5p^1^, Tl: 5d^10^6s^2^6p^1^, and N: 2s^2^2p^3^.

After the convergence test, in the vector K space, we took 1200 eV as the cut-off energy for the plane-wave expansion, which was large enough to obtain fine convergence. The size of the simulation model is listed in Table 1, and we took the experimental values as the original data. According to the Monkhorst-Pack scheme [29], 8 × 8 × 8 k-point meshes were constructed in the Brillouin zone integrations. All lattice constants used for structure optimization were experimental values. The lattice model and the atomic position in the lattice were optimized by the Broyden-Fletcher-Goldfarb-Shanno (BFGS) algorithm to determine the structure with the lowest energy [30,31,32,33]. The tolerances for geometry optimization were as follows: total energy within 5 × 10^−6^ eV/atom, maximum ionic Hellmann-Feynman force within 0.01 eV/Å, maximum stress within 0.02 GPa, and maximum ionic displacement within 5 × 10^−4^ Å. When these conditions were achieved, convergence occurred. The deformation modes, anisotropy, elastic properties, thermal conductivity, and hardness were further analyzed on this basis.

### 2.2. Structure Properties

**Figure 1:**
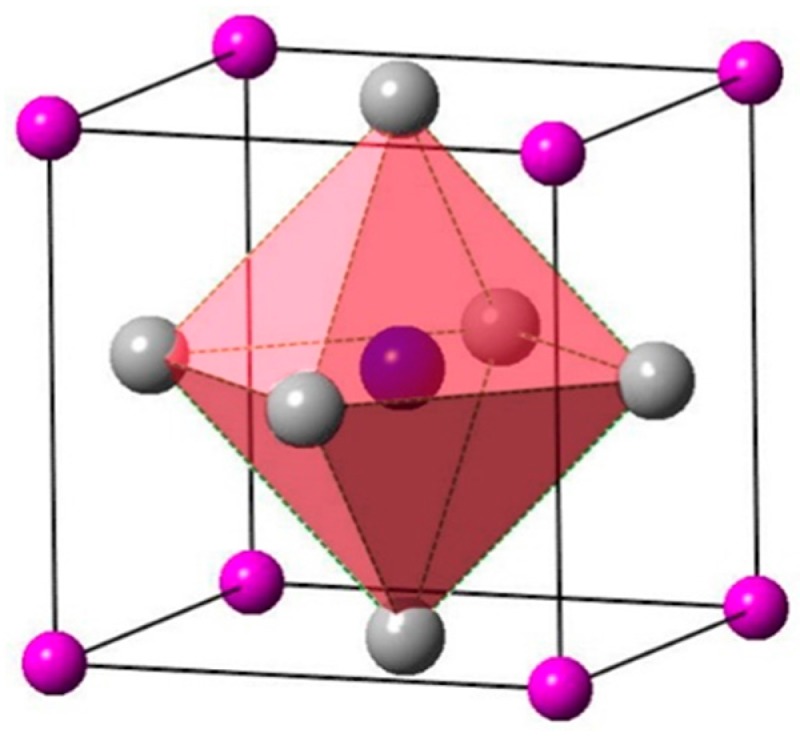
The anti-perovskite structure of Ti_3_AN with diagram of octahedron in which the Ti atoms located. 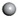: Ti atoms, 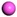: IIIA atoms, 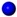: N atom (located in the body center).

The lattice structure of cermet (Ti_3_AN) is anti-perovskite and belongs to the space group $\text{Pm}\bar{3}\text{m}$, in which the A atom occupies the position (0, 0, 0) and the N atom occupies the position (0.5, 0.5, 0.5). In contrast to the ordinary perovskite structure, transition metals in the anti-perovskite structure are located at the corners of the octahedron cage [34], in other words, the Ti atom occupies the position (0, 0.5, 0.5). The lattice structure is shown in Figure 1.

The lattice parameters were obtained through the geometry optimization of the crystal structure at each degree of freedom, and the parameters matched the experimental values excellently. The elastic constants and the elastic modulus at the ground state of each crystal were calculated by linear fitting of the stress-strain curve. The bulk modulus and shear modulus were calculated using the Voigt and Reuss models, respectively [35,36].

(1) $$B_{V}=B_{R}=\left(C_{11}+2C_{12}\right)/3$$

(2) $$G_{V}=\left(C_{11}-C_{12}+3C_{44}\right)/5$$

(3) $$G_{R}=5\left(C_{11}-C_{12}\right)C_{44}/\left[4C_{44}+3\left(C_{11}-C_{12}\right)\right]$$

Using the extremum principle, Hill proved that the Voigt model and Reuss model are the upper and lower bounds of elastic constants, respectively. In addition, Hill suggested that the Voigt-Reuss-Hill (VRH) approximation is closer to the experimental results.

(4) $$B=\left(B_{V}+B_{R}\right)/2$$

(5) $$G=\left(G_{V}+G_{R}\right)/2$$

The Young’s modulus *E* and the Poisson’s ratios *ν* of the polycrystalline materials were further calculated based on the VRH values of the bulk modulus and shear modulus.

(6) E=9BG/(3B+G)

(7) ν=(3B−2G)/[2(3B+G)]

## 3. Results and Discussion

### 3.1. Structure Properties

First, the equilibrium lattice constants a (Å) for the anti-perovskite Ti_3_AN were calculated. Meanwhile, the elastic constants of Ti_3_AN were also calculated. The elastic constants of a crystal characterize its response to external stresses within the elastic limit, and the elastic constants of solids provide valuable information on their mechanical and dynamical properties. In particular, they can provide information on the stability and stiffness of the material [37]. The three independent elastic constants in cubic symmetry, i.e., *C*_11_*, C*_12_ and *C*_44_, were estimated by calculating the stress tensors upon applying strains to an equilibrium structure. We obtained the lattice constants, elastic constants, and elastic modulus of Ti_3_AN. The values are listed in Table 1.

The calculated lattice constants are close to the experimental ones [8,38] with small differences, since the LDA method gives a slight underestimation on the lattice constants. In addition, the bulk modulus *B* and shear modulus *G* were estimated using the VRH method. For a cubic system, the mechanical stability criterion [39] is expressed as $C_{11}>0$, $C_{44}>0$, $C_{11}>\left|C_{12}\right|$, and $C_{11}+2C_{12}>0$.

The elastic constants given in Table 1 conform to the criterion above, which indicates that these three structures are mechanically stable. Poisson’s ratio *ν* reflects the volume change in the materials under uniaxial deformation. When Poisson’s ratio equals 0.25 and 0.5, it represents the upper and lower limits of the central force solid, respectively. When it is equal to 0.5, the volume does not change under elastic deformation. The Poisson’s ratios of the three compounds are between 0.25 and 0.5, their binding force belongs to the central force [40]. It can be seen that the three compounds have large Poisson’s ratios (>0.3) and not too large Young’s moduli (<300 GPa) and shear moduli. Therefore, we can expect that the three compounds have comparatively low hardness. In addition, this prediction will be confirmed according to the results in our later analyses.

According to Pugh’s criterion [40], when *G/B* < 0.56, this kind of material generally exhibits toughness, when *G/B* > 0.56, it generally exhibits brittleness. The values shown in Table 1 indicate that the three compounds are tough materials.

*B/C*_44_ is taken as the basis for judging the lubrication performance of materials. The larger *B/C*_44_, the better the lubrication performance [41]. Table 1 reveals that all three compounds have good lubrication performance and are all good lubricants. As is known, a lubricant requires a certain tenacity, and according to the values of *G/B* in Table 1, all three compounds have high tenacity. As seen in Table 1, the Cauchy pressure *C*_12_*-C*_44_ is larger than zero. If the interatomic forces can be described by a potential that only depends on the distance between atoms and if all atoms of an unstrained crystal occupy the centers of inversion symmetry in the lattice, the Cauchy pressure vanishes, and *C*_12_ *= C*_44_. This form of the potential excludes torsional, or bending, forces (present in covalent crystals) and interatomic forces, which vary with the atomic volume (present in metals) [42]. If the Cauchy pressure is positive (negative), the material is ductile (brittle).

The influence of microcracks and lattice distortions is typically considered when studying the mechanical properties of materials. Anisotropy of the elastic properties is usually the condition responsible for the formation of the two abovementioned factors. Therefore, research into elastic anisotropy is beneficial for improving the mechanical durability of materials. In the lattice of Ti_3_AN, metallic bonds among metal atoms have no directionality, whereas the bonds between Ti atoms and N atoms are parallel to the basis vectors of the lattice with strong directionality. In addition, this is the cause of anisotropy in the elastic modulus. Therefore, the elastic properties along the face diagonal and body diagonal may significantly differ.

Shivakumar and Ranganathan introduced the elastic anisotropy index *A^U^* [43], which is applicable to all crystals, to quantitatively study the anisotropy of a monocrystal,

(8) $$A^{U}=5G_{V}/G_{R}+B_{V}/B_{R}-6\geq 0,$$

where *B_V_* and *B_R_* are the bulk moduli of the Voigt and Reuss models, respectively, and *G_V_* and *G_R_* are the shear moduli of the Voigt and Reuss models, respectively. *A^U^* = 0 indicates the isotropy of a monocrystal. An *A^U^* deviating from 0 results in greater anisotropy in the materials. Moreover, Chung and Buessem [44] proposed the concept of fractional anisotropy ratios, which are defined as follows:

(9) $$A^{B}=\left(B_{V}-B_{R}\right)/\left(B_{V}+B_{R}\right),$$

(10) $$A^{G}=\left(G_{V}-G_{R}\right)/\left(G_{V}+G_{R}\right).$$

These ratios were used to evaluate the degree of anisotropy of the bulk modulus and the shear modulus. *A^B^* = 0 and *A^G^* = 0 indicate that the materials are isotropic. Meanwhile, *A^B^* = 1 and *A^G^* = 1 indicate the greatest possible elastic anisotropy. *A^E^* was defined as the Young’s anisotropic factor, and the formula is as follows:

(11) $$A^{E}=\left(E_{V}-E_{R}\right)/\left(E_{V}+E_{R}\right).$$

All of the anisotropy values of Ti_3_AN are listed in Table 2. The calculated results show that all anisotropy indices *A^U^* of the three compounds are larger than 0, which indicates the anisotropy of the elastic properties, and the degree of anisotropy inTi_3_InN is significantly larger compared with that in Ti_3_AlN and Ti_3_TlN. The Young’s modulus and shear modulus of Ti_3_AN are all anisotropy.

**Figure 2:**
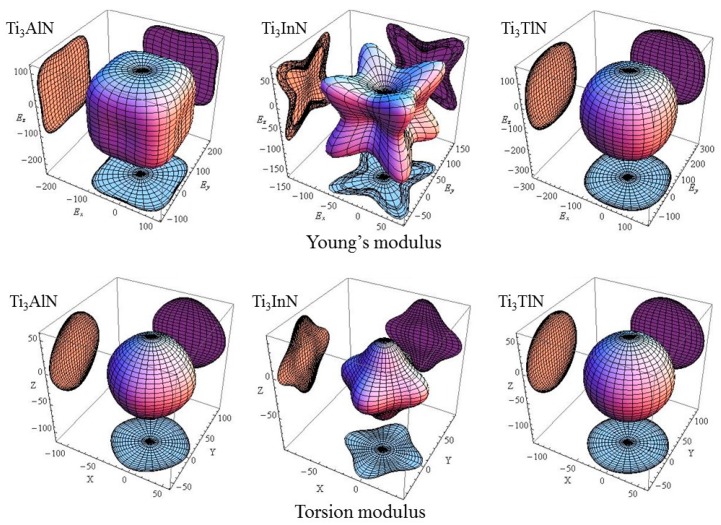
Young’s and torsion modulus of Ti_3_AN in full space. (A = Al, In and Tl).

To observe the change in elastic modulus of each cell in different crystal orientations, the Young’s modulus of Ti_3_AN in full space is shown in Figure 2. In addition, the calculation formula is as follows [45]:

(12) $$E^{-1}=S_{11}-2\left(S_{11}-S_{12}-S_{44}/2\right)\left(l_{1}^{2}l_{2}^{2}+l_{2}^{2}l_{3}^{2}+l_{3}^{2}l_{1}^{2}\right).$$

Here, *S_ij_* is the elastic compliance coefficient; Ti_3_AN has three independent elastic compliances, which are *S*_11_ = *S*_22_ = *S*_33_, *S*_44_ = *S*_55_ = *S*_66_, and *S*_12_ = *S*_13_ = *S*_23_, and the rest are zero. In addition, *l*_1_, *l*_2_, and *l*_3_ are the direction cosines ($l_{1}=\sin\theta \cos\varphi ,l_{2}=\sin\theta \sin\varphi ,\mathrm{and}l_{3}=\cos\theta )$, where a larger $\left(l_{1}^{2}l_{2}^{2}+l_{2}^{2}l_{3}^{2}+l_{3}^{2}l_{1}^{2}\right)$ leads to a larger *E*. However, the shear modulus is determined by two factors. One is the force-exerting plane, and the other is the force-exerting direction. The latter has infinite possibility on the plane. Therefore, the shear modulus cannot be plotted in 3D space. However, the torsion modulus can be plotted, which is the average shear modulus over all possible directions. Figure 2 shows the torsion modulus [46], and the calculation formulas are as follows:

(13) $$T^{-1}=S_{44}+4\left[\left(S_{11}-S_{12}\right)-S_{44}/2\right]\left(l_{1}^{2}l_{2}^{2}+l_{2}^{2}l_{3}^{2}+l_{3}^{2}l_{1}^{2}\right),$$

The calculated values of *E* and *T* in the direction of [100], [110], and [111] are listed in Table 2.

In Figure 2, the plots of the Young’s modulus and torsion modulus of Ti_3_AlN, Ti_3_InN, and Ti_3_TlN are remarkably different in full space. For Ti_3_AlN, the plot of the Young’s modulus is similar to a cube and shows the minimum Young’s modulus in the [100] direction, followed by the [110] and [111] directions. The plot of the torsion modulus of Ti_3_AlN looks like an octahedron, indicating that Ti_3_AlN has a minimum torsion modulus in the [111] direction, followed by the [110] and [100] directions. For Ti_3_InN, the diagrams are distinct. It is obvious that Ti_3_InN has a maximum Young’s modulus and minimum torsion modulus in the [111] direction, followed by the values in the [110] and [100] direction, respectively. For Ti_3_TlN, the Young’s modulus and torsion modulus have relatively similar diagrams, which indicates slight anisotropy. In general, the anisotropy of Ti_3_AN increases in the order of Ti_3_TlN→Ti_3_AlN→Ti_3_InN.

### 3.2. Electronic Structures

**Figure 3:**
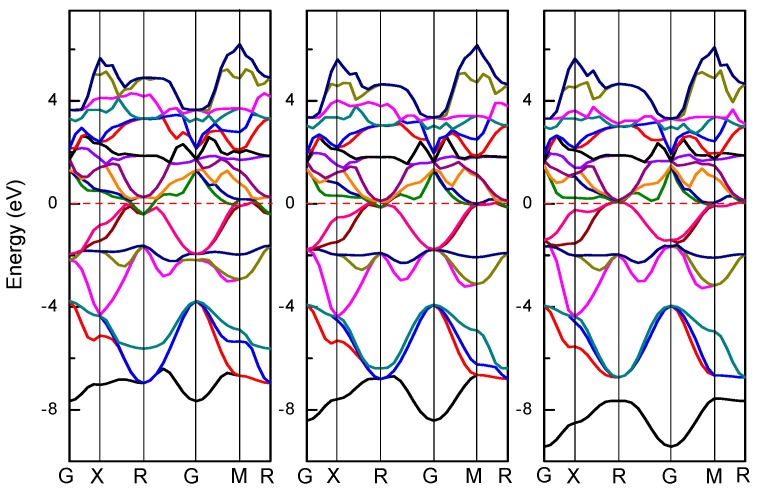
Energy band structures of Ti_3_AlN, Ti_3_InN, and Ti_3_TlN.

To understand the electronic structure, the energy band structure of Ti_3_AlN, Ti_3_InN, and Ti_3_TlN were calculated. The band structures of Ti_3_AN are depicted in Figure 3. The red dotted lines represent the Fermi energy levels. The characteristics of the electrons near the Fermi surface primarily determine the properties of the materials. As shown in Figure 3, the energy band structures of the three compounds are very similar. In these band structures, which have no energy gaps, the excitation of energy bands from the valence band to the conduction band occurs across the Fermi level, indicating the existence of free electrons. Therefore, all three compounds have metal-like conductive properties.

**Figure 4:**
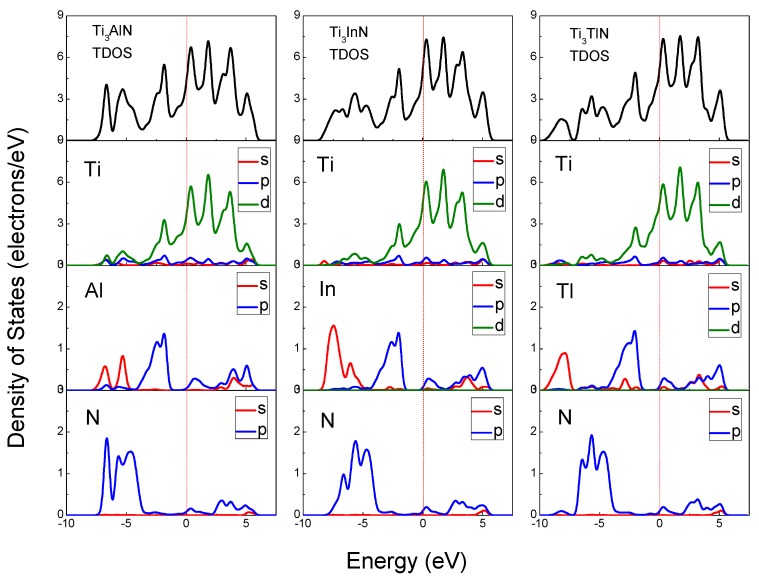
Density of states of Ti_3_AlN, Ti_3_InN, and Ti_3_TlN.

The total density of states (TDOS) and partial density of states (PDOS) of Ti_3_AlN, Ti_3_InN, and Ti_3_TlN are shown in Figure 4 and reveal the composition of the electronic states in the energy band structure. The origin of the band structure spectra is due to A s, A p, and N p states, with contributions from Ti s, Ti p, and Ti d states in the energy range of −9.2 to 6.5 eV. From the Fermi level to 6.5 eV, the band structure is originally derived from the A p and Ti d states. The Ti d states remain the majority at the Fermi level; therefore, the main part of the electrical conductivity is due to the d electrons of the transition metal Ti. On the left side of the Fermi level, the band structure spectra are mainly derived from Ti d states hybridized with A s, A p, A d, N s, and N p electrons. The peaks located near −5 eV and 2 eV correspond to the Ti 3d and N 2p states. The hybridization of the Ti d states with the N s and N p states suggests a strong covalent bonding contribution in the Ti_3_AN compounds, which corresponds to the Ti-N bonds in the lattice. The density of states of the hybridized peaks near −5 eV is mainly attributed to the 2p states of the N atoms, whereas the d states of the Ti atoms contributed less. For the peaks near 2 eV, the d states of the Ti atoms have major contributions, whereas the contributions from the N atoms are small. It is clearly observed that all PDOSs of the Ti atoms are significantly larger than those of the N atoms for the hybridized peaks near −5 eV and 2 eV, and the calculated Hirshfeld analysis shows that the Hirshfeld charge of N atoms for all the three compounds is −0.34, in contrast, the Hirshfeld charge of Ti atoms is positive (0.04 for Ti_3_AlN, 0.07 for Ti_3_InN, and 0.06 for Ti_3_TlN), thus, the Ti-N bonds exhibit some ionicity [47]. And in the Mulliken’s population analysis, the bond population of Ti-N bonds are all positive (as shown in Table 3), indicating the covalency of the Ti-N bonds [48]. In conclusion, the electronic structures of Ti_3_AN have been proven to be a mixture of metallicity, covalency, and ionicity. Therefore, these compounds present the characteristics of both metals and ceramics, such as electrical conductivity and oxidation resistance.

### 3.3. Deformation Modes

**Figure 5:**
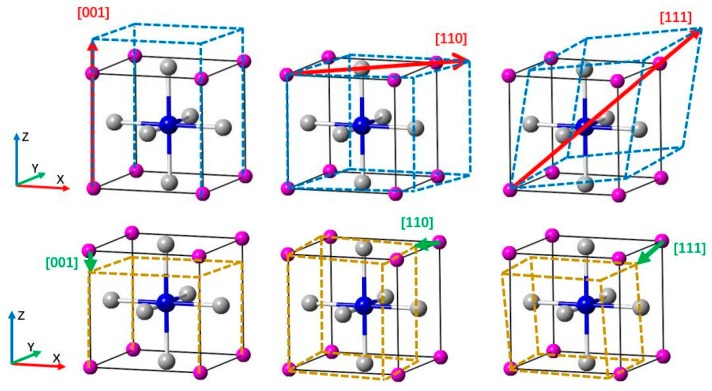
The diagram of tension and compression.

**Figure 6:**
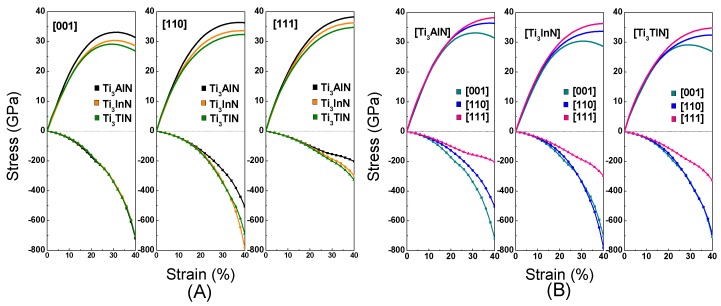
Stress-strain curves of tension and compression. (**A**) The tension and compression stress-strain curves in the [001], [110], and [111] crystal orientations for Ti_3_AN; (**B**) The anisotropy in the stress-strain variation of Ti_3_AN in different crystal orientations. (The smooth lines represent the tension and the lines with symbols represent the compression and the minus sign represents the magnitude of the compressive stress).

We calculated the tensile stress-strain curve in the [001], [110], and [111] crystal orientations to study the deformation mechanism of Ti_3_AN. With tensile loading, the materials usually experience a variation from the elastic region to the unstable region and finally fracture as the strain increases. When the elastic deformation reaches its limit, a turning point appears in the tensile curve, which means that the atoms break away from the pull of adjacent atoms and spontaneously slide from the original lattice position to a new one to attain a minimum total energy in the system [49]. The diagrams of tension and compression are shown in Figure 5. In addition, the stress-strain curves are shown in Figure 6. Notably, these figures are plotted with data calculated by the LDA method, which is used hereafter.

Figure 6A shows the tension and compression stress-strain curves in the [001], [110], and [111] crystal orientations for Ti_3_AN. Notably, the Ti_3_AN samples in the [001] orientation show a variation from the elastic region to the unstable region and then fracture. The maximal tensile stresses, defined as the ideal tensile strength, equal 33.12 GPa, 30.37 GPa, and 29.12 GPa in the [001] crystal orientation for Ti_3_AlN, Ti_3_InN, and Ti_3_TlN, corresponding to a strain of 32%, 30%, and 30%, respectively. Ti_3_AlN presents the maximal tensile strength along the [001], [110], and [111] directions, and Ti_3_AlN has a relatively broader elastic region in the [001] crystal orientation compared with Ti_3_InN and Ti_3_TlN. Thus, we can conclude that Ti_3_AlN possesses the best ideal strength among the three compounds.

To achieve an increase in the compressive strain, the compressive stress was increased continuously. The three compounds show striking similarities when compressed along the [001] crystal orientation. However, the curves show differences when the compounds are compressed along the [110] and [111] crystal orientation. Ti_3_InN shows the most distinct shape under tension and compression.

Figure 6B shows the anisotropy in the stress-strain variation of Ti_3_AN in different crystal orientations. The pattern shows differences in the shape of the curve, which means that the three compounds have different performances under tensile stress in the [001], [110], and [111] crystal orientations. In addition, the three compounds show the best mechanical performance when stretched along the [111] crystal orientation. The order is τ[111] > τ[110] > τ[001]. We suppose the cause of this behavior is the stretching along the [111] crystal orientation elongating more bonds. However, when we apply pressure to the three compounds in the three crystal orientations, the compression performance in the [111] orientation is relatively poor. In the [111] crystal orientation, the same compression can cause greater deformation. As is known, crystal slippage often occurs in the most densely packed crystal orientation, and as [111] is the most densely packed crystal orientation, this may be the reason for the poor performance in the [111] orientation under compression.

To produce the same strain, the pressure should be much greater than the tension, and the pressure tends to increase with an increase in strain. The chemical bonds in these compounds are stable, and there is a certain distance between the atoms in the compounds. Compressing the distance is more difficult than stretching the distance, which may be the cause of the phenomenon.

The electrons density distribution is a basic calculation quantity and can be obtained from calculations based on DFT. The electron density difference can be derived from the electrons density distribution. The electron density difference can express the redistribution of electrons after the construction of a system with atoms. The bonding situation can be obtained intuitively from the electron density difference. In addition, the electron density difference can further explain the tensile deformation mechanism of materials in the angle of electron transfer. The computational formula is as follows [50]:

(14) $$\Delta \rho =\rho \left(Ti_{3}AN\right)-\left[\rho \left(Ti\right)+\rho \left(A\right)+\rho \left(N\right)\right],$$

where $\rho \left(Ti_{3}AN\right)$ is the electron density of the compound system, ρ(Ti), ρ(A) and ρ(N) represent the electron density of Ti atoms, A-group atoms and N atoms in the free state, respectively.

**Figure 7:**
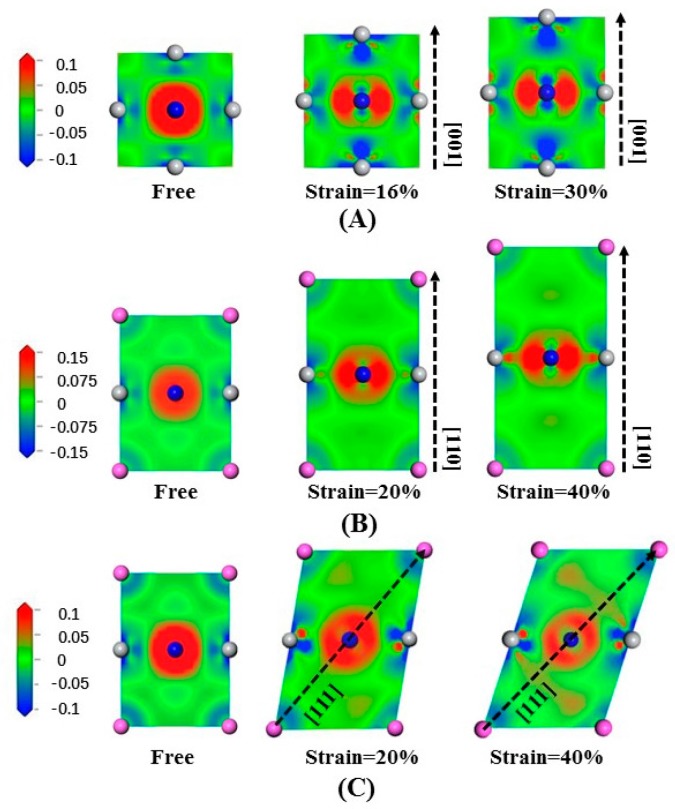
Electron density difference of Ti_3_AlN: (**A**) stretched along [001] in the slice of (200); (**B**) stretched along [110] in the slice of $\left(1\bar{1}0\right)$; (**C**) stretched along [111] in the slice of $\left(1\bar{1}0\right)$.

Figure 7A illustrates the electron density difference in Ti_3_AlN in slice along the (200) crystal plane stretched along [001]. We notice that the Ti-Ti bonds undergo slight changes. In contrast, the electron density distribution of the Ti-N bonds undergoes a dramatic change. Stretching in the [001] crystal orientation strengthens the effect of electron transfer by the Ti-N bonds in the (200) crystal plane, either along the direction parallel to the stretching direction or along the direction perpendicular to the stretching direction. For the Ti-N bonds along the stretching direction, the electrons tends to be away from the Ti atoms, while for the Ti-N bonds perpendicular to the stretching direction, the electrons tends to be close to the N atoms.

Figure 7B illustrates the electron density difference of Ti_3_AlN in slice along the $\left(1\bar{1}0\right)$ plane stretched along [110] in order to illustrate the electron density arrangement. The Ti-Al bonds undergo slight changes, while the Ti-N bonds changed greatly. Stretching strengthens electron transfer in the transverse direction, and the electrons tend to keep close to the N atoms in the direction perpendicular to the stretching.

The electron density difference of tensile-deformed Ti_3_AlN in slice along the $\left(1\bar{1}0\right)$ plane stretched along [111] is presented in Figure 7C to illustrate the electron density arrangement. We can clearly notice the deformation of the cell under tensile strain. In addition, electrons transfer along the Ti-Al bonds in the stretching direction is strengthened, while electrons transfer in the perpendicular direction is weakened. Ti-N bonds maintain their toughness during the tensile stress process.

**Figure 8:**
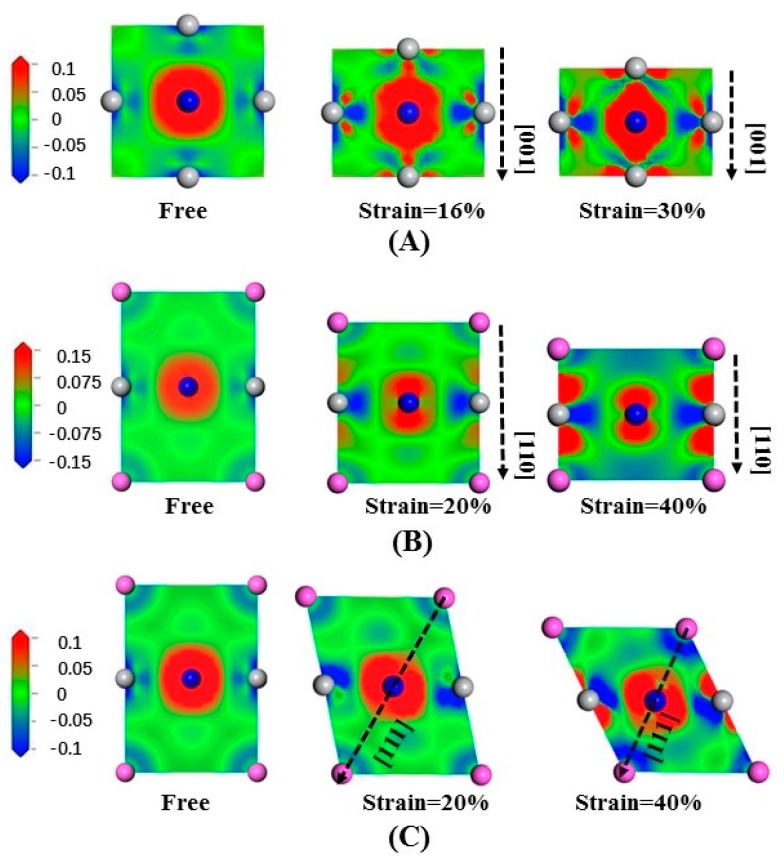
Electron density difference of Ti_3_AlN: (**A**) compressed along [001] in the slice of (200); (**B**) compressed along [110] in the slice of $\left(1\bar{1}0\right)$; (**C**) compressed along [111] in the slice of $\left(1\bar{1}0\right)$.

Figure 8 shows the electron density difference of Ti_3_AlN under compression, where (A) is the slice of (200) compressed along [001]; (B) is the slice of $\left(1\bar{1}0\right)$ compressed along [110]; and (C) is the slice of $\left(1\bar{1}0\right)$ compressed along [111]. In Figure 8A, in the direction perpendicular to compression, the electrons tend to stay away from the Ti atoms. In Figure 8B, in the direction parallel to compression, the electrons tend to gather near the N and Ti atoms, but in the direction perpendicular to compression, the electrons tend to be away from the Ti atoms. When compressed along [111], the Ti-N bonds are greatly changed, and the electrons tend to stay away from the Al and Ti atoms in the direction parallel to the compression and be close to the N atoms in the direction perpendicular to the compression, as shown in Figure 8C.

By analyzing the changes in the bonds during the deformation process, taking the same standard scale for both tension and compression, we find that when the same strain is obtained, the electron density difference changes immensely under compression. This may explain why compression requires much greater stress than tension. The features are the same for Ti_3_InN and Ti_3_TlN under tensile deformation, which is not presented here for brevity.

### 3.4. Hardness

Hardness is an important physical quantity for characterizing the resistance of solid materials to elastic and plastic deformation. In general, materials with greater hardness have better wear resistance. In addition, materials with low hardness may act as lubricants. In other words, hardness is an important indicator of the wear resistance of materials. Due to Ti_3_AN simultaneously containing metallic bonding, ionic bonding, and covalent bonding, we can obtain the hardness of Ti_3_AN by the following formulas [51,52,53]:

(15) $$H_{V}={\left[\prod_{\mu }{\left(H_{V}^{\mu }\right)}^{n^{\mu }}\right]}^{1/\sum^{\text{​}}n^{\mu }},$$

(16) $$H_{V}^{\mu }\left(GPa\right)=740P^{\mu }{\left(V_{b}^{\mu }\right)}^{-5/3},$$

(17) $$V_{b}^{\mu }={\left(d^{\mu }\right)}^{3}/\sum_{V}\left[{\left(d^{V}\right)}^{3}N_{b}^{V}\right],$$

in which $H_{V}$ is the hardness, $H_{V}^{\mu }$ is the hardness of the μ bond, $P^{\mu }$ is the calculated Mulliken population, $V_{b}^{\mu }$ is the volume of the μ bond, $d^{\mu }$ is the bond length of the μ bond, $n^{\mu }$ is the number of μ bonds.

The population number and bond length of Ti_3_AN were calculated, and the values are listed in Table 3. In addition, the calculated hardness of the three compounds is also listed in Table 3. Ti_3_InN has the lowest hardness compared with Ti_3_AlN and Ti_3_TlN, which is 6.87 GPa. By analyzing the bonds in the Ti_3_AN lattice, the bond length, the population and the bond volume of the Ti-N bonds, no significant difference was found in the three compounds. When examining the values of the metallic bonds, we can clearly see that the bond lengths, populations, and bond volumes are similar, except for the population of the Ti-Ti bonds in Ti_3_InN. The population of the metal-metal bond in Ti_3_InN is obviously low compared with the ones in Ti_3_AlN and Ti_3_TlN, which is 0.33. We suppose this phenomenon to be the reason for the anomalously low bond hardness of the Ti-Ti bonds in Ti_3_InN, which leads to a reduction in the hardness of Ti_3_InN. In other words, the high bond population of Ti_3_AN suggests high hardness. The calculated hardness is 10.73 GPa and 11.14 GPa for Ti_3_AlN and Ti_3_TlN, respectively. We can see that the calculated results for Ti_3_AlC are in agreement with the experimental data, and Ti_3_AlC has similar lattice structure and bonds with Ti_3_AN, thus, our calculations and hardness formulas for Ti_3_AN are reliable.

### 3.5. Anisotropy of the Minimum Thermal Conductivity

Lattice vibrations determine many physical properties of a crystal. In addition, lattice vibrations can be reflected by the phonon system. Acoustic waves are key physical quantities with significant function in studying the thermal conductivity of a material. We calculated the wave speed of the transverse and longitudinal acoustic waves for Ti_3_AN in the [100], [110], and [111] crystal orientations. Table 4 shows the results, and the calculation formulas are as follows [55]:

(18) $$v_{l}\left[100\right]=\sqrt{C_{11}/\rho },\;v_{t1}\left[010\right]=v_{t2}\left[001\right]=\sqrt{C_{44}/\rho };$$

(19) $$v_{l}\left[110\right]=\sqrt{\left(C_{11}+C_{12}+2C_{44}\right)/2\rho },\;v_{t1}\left[1\bar{1}0\right]=\sqrt{\left(C_{11}-C_{12}\right)/2\rho },\;v_{t2}\left[001\right]=\sqrt{C_{44}/\rho };$$

(20) $$v_{l}\left[111\right]=\sqrt{\left(C_{11}+2C_{12}+4C_{44}\right)/3\rho },\;v_{t1}\left[11\bar{2}\right]=v_{t2}=\sqrt{\left(C_{11}-C_{12}+C_{44}\right)/3\rho }.$$

where *C_ij_* is the elastic constant and ρ is the density. For a cubic crystal system, there are two dispersion curves between Γ[000] and Χ[100] in the phonon spectrum, which are correlated to a non-degenerate longitudinal acoustic branch and a doubly degenerate transverse acoustic branch, respectively [55]. Therefore, the two transverse acoustic waves along [100] have the same wave speed. The acoustic wave in [111] is similar to that in [100]. In [110], all of the phonon dispersion curves are in the non-degenerate state, and thus the two transverse acoustic waves propagate at different wave speeds. The distributions of the two transverse acoustic waves in the phonon spectrum significantly differ. The calculated results of CaO are also shown in Table 4, and the calculated wave speed is in good agreement with experimental values, indicating that the calculation method is reliable.

The acoustic wave speed closely relates to the thermal conductivity of a material. At high temperature, the thermal conductivity decreases with increasing temperature [55]. Therefore, determining the minimum value of the thermal conductivity at high temperature is important for exploring the application of materials under extreme conditions. We calculated the thermal conductivity of polycrystalline Ti_3_AN by the Clark model and Cahill model [55,57].

(21) $$\mathrm{Clark Model}:\;K_{min}=0.87k_{B}M_{a}^{-2/3}E^{1/2}\rho^{1/6},$$

(22) $$\mathrm{Cahill Model}:\;K_{min}=k_{B}p^{2/3}\left(v_{l^{\prime }}+2v_{t^{\prime }}\right)/2.48,$$

(23) $$v_{t^{\prime }}=\sqrt{G/\rho },v_{l^{\prime }}=\sqrt{\left(B+4G/3\right)/\rho }.$$

where *E* is the Young’s modulus, *ρ* is the density, $k_{B}$ is Boltzmann’s constant, $M_{a}=\left[M/\left(n\cdot N_{A}\right)\right]$ is the average mass for an atom in the lattice, M is the molar mass of the molecule, n is the number of atoms, $N_{A}$ is Avogadro’s constant, p is the number of atoms per unit volume, and $v_{l^{\prime }}$ and $v_{t^{\prime }}$ are the wave speeds for the transverse acoustic wave and longitudinal acoustic wave, respectively. We calculated the lower limit of the lattice thermal conductivity based on the two models, and the results are shown in Table 5. The high-temperature thermal conductivity decreased in the order of Ti_3_AlN→Ti_3_TlN→Ti_3_InN. The accuracy of the calculated results for ZrO_2_ becomes quite satisfactory with the experimental data in Table 5, which supplies the safeguard for the following studies.

In contrast to the Clark model, the Cahill model is based on the wave speed of the lattice vibration. In the formula, $v_{l^{\prime }}$ and $v_{t^{\prime }}$ correspond to the acoustic wave speeds along the crystal orientation. Therefore, we can calculate the thermal conductivity of the lattice in different lattice orientations. The alternative formula is as follows [57]:

(24) $$K_{min}=k_{B}p^{2/3}\left(v_{l}+v_{t1}+v_{t2}\right)/2.48.$$

In this paper, we calculated the minimum thermal conductivity of the three compounds in the [100], [110], and [111] direction. The detailed results are presented in Table 6.

The difference in the wave speed of the acoustic wave along the different crystal orientations indicates the anisotropy in the thermal conductivity. As shown in Table 6, the value of $K_{min}\left[100\right]$, $K_{min}$[110] and $K_{min}$[111] are quite different; therefore, Ti_3_AN has anisotropic thermal conductivity. For a crystal with isotropic thermal conductivity, $K_{min}\left[100\right]$ = $K_{min}$[110] = −$K_{min}$[111] = $K_{min}$. Thus, we can determine the degree of anisotropy in the thermal conductivity by comparing $K_{min}$(avg) = ($K_{min}\left[100\right]$ + $K_{min}$[110] + $K_{min}$[111])/3 and $K_{min}$[hkl]. The difference between $K_{min}\left[100\right]$ and $K_{min}$(avg), $K_{min}$[110], and $K_{min}$(avg), and $K_{min}$[111] and $K_{min}$(avg) are 0.36%, 0.14%, and 0.43% for Ti_3_AlN, respectively; those for Ti_3_InN are 0.53%, 0.74%, and 0.21%, respectively; and those for Ti_3_TlN are 0.1%, 0, and 0.19%, respectively. Thus, the degree of anisotropy in the thermal conductivity shows a general increase in the order of Ti_3_TlN→Ti_3_AlN→Ti_3_InN. All three compounds have relatively low thermal conductivity, and the thermal conductivity meets the requirements of heat-insulator materials.

## 4. Conclusions

The elastic anisotropy, electronic structures, hardness, and minimum thermal conductivity of anti-perovskite Ti_3_AlN, Ti_3_InN and Ti_3_TlN were investigated by the pseudo potential plane-wave method based on DFT. All of the compounds are elastic anisotropic. The anisotropy of Ti_3_InN is significantly larger than that of Ti_3_AlN and Ti_3_TlN, and in the order of Ti_3_TlN < Ti_3_AlN < Ti_3_InN.

In the process of deformation, the states of the bonds changed by a certain degree. Ti_3_AlN has relatively good mechanical properties. Shown by the population analysis, Ti-N bonds are the strongest bonds, and the strength of the Ti-N bonds is responsible for the structural stability of the three compounds.

The electronic structures of the three compounds show that there are no band gaps between the conduction and the valence band, indicating that the three compounds exhibit metallicity in the ground state, which is a typical metallic feature. The density of states of all valence orbital electrons of the Ti atoms, A group elements, and N atoms overlapped near the Fermi energy level, revealing that the three compounds show a mixture of metallicity, covalency, and ionicity.

The minimum thermal conductivity at high temperature is very small and decreases in the sequence of Ti_3_AlN→Ti_3_TlN→Ti_3_InN. These materials can be used as heat-insulator materials.

## Figures and Tables

**Table 1:** Calculated lattice constants *a*/Å, density *ρ*/g·cm^−3^, elastic constants *C_ij_*/GPa, bulk modulus *B*/GPa, shear modulus *G*/GPa, *G/B*, Young’s modulus *E*/GPa, Poisson’s ratio *ν*, *B/C*_44_ and *C*_12_*-C*_44_. LDA: local density approximation.

Parameters	Ti_3_AlN			Ti_3_InN			Ti_3_TlN		
-	LDA	Ref. [23]	Expt.	LDA	Ref. [23]	Expt.	LDA	Ref. [23]	Expt.
*a*, *b*, *c*	4.050	4.051	4.112 [36]	4.114	4.116	4.190 [37]	4.122	4.124	4.191 [37]
*ρ*	4.636	-	-	6.525	-	-	8.619	-	-
*C*_11_	239.07	239.94	-	196.48	189.85	-	258.97	256.36	-
*C*_12_	159.09	156.60	-	172.69	171.05	-	145.25	138.44	-
*C*_44_	57.63	57.91	-	45.62	49.68	-	62.38	63.76	-
*B*	185.75	184.38	-	180.62	177.32	-	183.16	177.75	-
*G*	49.78	50.76	-	26.75	25.93	-	60.11	61.79	-
*E*	137.10	139.47	-	76.48	74.19	-	162.56	166.13	-
*G/B*	0.27	-	-	0.15	-	-	0.33	-	-
*ν*	0.377	-	-	0.43	-	-	0.35	-	-
*B/C*_44_	3.22	-	-	3.96	-	-	2.93	-	-
*C*_12_*-C*_44_	101.46	-	-	127.07	-	-	82.87	-	-

Expt.: data from experiments.

**Table 2:** The calculated *A^U^*, *A^E^*, *A^G^*, *A^B^*, *E*_[001]_, *E*_[110]_, *E*_[111]_, *T*_[100]_, *T*_[110]_ and *T*_[111]_ of Ti_3_AN.

Species	*A^U^*	*A^E^*	*A^G^*	*A^B^*	*E*_[001]_	*E*_[110]_	*E*_[111]_	*T*_[100]_	*T*_[110]_	*T*_[111]_
Ti_3_AlN	0.162	0.015	0.016	0	111.9	142.5	152.9	57.6	54.6	53.9
Ti_3_InN	2.515	0.192	0.201	0	34.9	76.3	108.5	45.6	33.7	31.6
Ti_3_TlN	0.010	0.001	0.001	0	154.6	164.5	167.2	62.4	61.6	61.5

**Table 3:** Bond types, number of bonds in unit crystal, bond length, population, bond volume (Å^3^), bond hardness and hardness (GPa).

Species	Bond	Nb	Length	Population	Vb	Hvb	Hv	$H_{v}^{expt.}$
Ti_3_AlN	Ti-N	3	2.025	0.51	5.78	20.26	10.73	-
	Ti-Al	3	2.864	0.81	16.36	5.69		
Ti_3_InN	Ti-N	3	2.057	0.60	6.06	22.03	6.87	-
	Ti-Ti	3	2.909	0.33	17.15	2.14		
Ti_3_TlN	Ti-N	3	2.061	0.61	6.10	22.18	11.14	-
	Ti-Ti	3	2.915	0.87	17.25	5.59		
Ti_3_AlC	Ti-C	3	2.052	0.62	6.02	17.02	11.27	7.8~12.5 [54]
	Ti-Al	3	2.902	0.84	23.04	5.52		

**Table 4:** Anisotropy acoustic wave speed of Ti_3_AN (km·s^−1^).

Species	[100]		[110]			[111]	
	$v_{t1}\left[010\right],v_{t2}\left[001\right]$	$v_{l}\left[100\right]$	$v_{t1}\left[1\bar{1}0\right]$	$v_{t2}\left[001\right]$	$v_{l}\left[110\right]$	$v_{t1}\left[11\bar{2}\right],v_{t2}$	$v_{l}\left[111\right]$
Ti_3_AlN	3.28	7.38	2.94	3.53	7.44	3.15	7.53
Ti_3_InN	2.02	5.76	1.35	2.64	5.94	1.88	6.08
Ti_3_TlN	2.64	5.53	2.57	2.69	5.54	2.61	5.56
CaO_cal_	4.70	8.10	7.03	4.70	7.94	4.88	7.89
CaO_exp_ [56]	4.94	8.21	7.02	4.94	8.19	4.96	8.18

**Table 5:** Average mass (g) of atoms, transverse acoustic wave and longitudinal wave speed (km·s^−1^), atomic number per unit volume, and minimum high-temperature thermal conductivity (W·m^−1^·K^−1^) for polycrystalline Ti_3_AN.

Species	Clark		Cahill				$K_{min}^{expt.}$
	Ma (10^−23^)	$K_{min}$	$v_{t^{\prime }}$	$v_{l^{\prime }}$	P × 10^28^	$K_{min}$	
Ti_3_AlN	6.13	1.17	3.28	7.34	7.53	1.38	-
Ti_3_InN	9.05	0.71	2.02	5.76	7.18	0.94	-
Ti_3_TlN	12.03	0.90	2.64	5.51	7.14	1.04	-
ZrO_2_	6.83	1.74	4.31	8.14	9.45	1.94	2.2 [58]

**Table 6:** Minimum thermal conductivity (W·m^−1^·K^−1^) at high temperature in different crystal orientation.

Species	$K_{min}$[100]	$K_{min}$[110]	$K_{min}$[111]	$K_{min}$(avg)
Ti_3_AlN	1.384	1.381	1.373	1.379
Ti_3_InN	0.943	0.955	0.946	0.948
Ti_3_TlN	1.036	1.035	1.033	1.035
